# Editorial: Wearable Devices for Cardiac Rhythm Monitoring

**DOI:** 10.3389/fcvm.2022.951769

**Published:** 2022-06-21

**Authors:** David Duncker, Emma Svennberg

**Affiliations:** ^1^Hannover Heart Rhythm Center, Department of Cardiology and Angiology, Hannover Medical School, Hannover, Germany; ^2^Department of Medicine Huddinge, Karolinska Institutet, Karolinska University Hospital, Stockholm, Sweden

**Keywords:** mobile health (mHealth), wearable device, atrial fibrillation, screening, rhythm monitoring

New wearable technologies for cardiac rhythm monitoring are gaining more importance in clinical routine in the field of cardiology and electrophysiology - by physicians as well as patients. These include, but are by far not restricted to, smartphone-based electrocardiogram (ECG) or photoplethysmograpy (PPG), finger-ECG, smartwatches, smart garments and more. This opens new horizons for mobile (m) Health-based patient care, mHealth-enhanced teleconsultations, but also mass screening for heart rhythm disorders.

The current Research Topic includes new research on these technologies covering methodological aspects on wearable single- and multiple-lead ECG or PPG devices as well as clinical implementation of digital devices (Figure 1).

**Figure 1:**
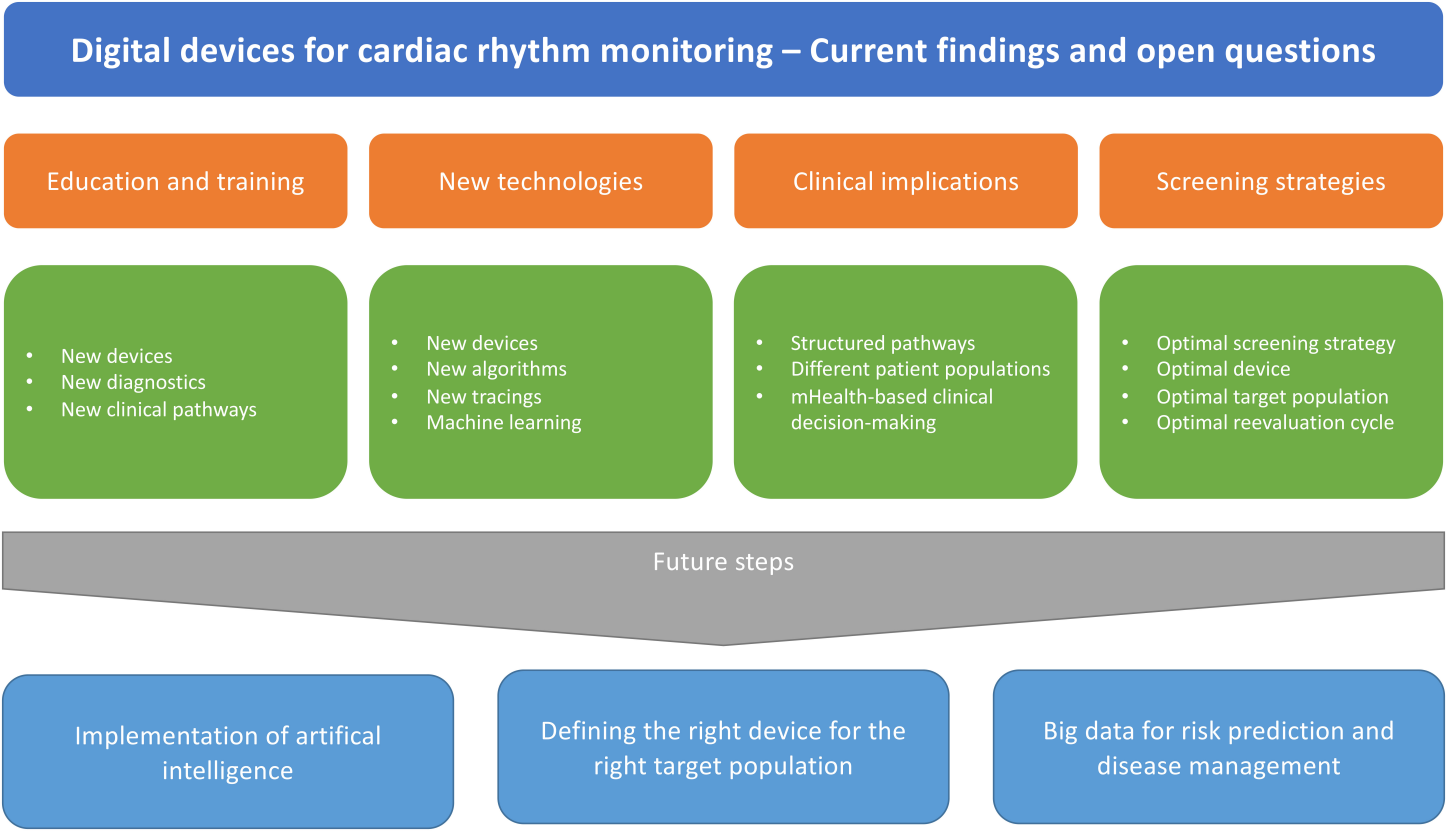
Clinical implementation of digital devices.

Xintarakou et al. present an elaborated review about smart wearables for monitoring and management of cardiac arrhythmias. The sensitivity and specificity of PPG-based devices in detecting AF is very good. Interpreting the PPG waveforms and tracings, however, requires some training (1). The INTERPRET-AF study by Gruwez et al. show that the accuracy of physicians interpreting PPGs is quite high and that using all available information from the PPG signal, the tachogram, the Poincaré plot and an automated algorithm increases the diagnostic accuracy and is comparable to a single lead ECG or 12-lead ECG. However, a call for training and education of PPG tracings and validity and limitations of interpretation should be made as this is rare in cardiological curricula, except in the recently updated curricula by the German Cardiac Society (2).

## New Devices

Comparative studies on different devices are rare. Abu-Alrub et al. compared recording quality of three single-lead smartwatches in 100 patients with atrial fibrillation vs. 100 patients in sinus rhythm. Diagnosing AF is possible using various ECG smartwatch models, but differences in diagnostic accuracy of the related automated algorithms were noted.

An electronic-textile-based ECG monitoring was evaluated by Teferra et al. showing an effective option for continuous cardiac monitoring implemented into textiles.

Combining digital devices with machine learning algorithms represents a unique opportunity for individualized approaches or early identification of patients at risk. Luongo et al. evaluated a machine learning algorithm using a single-lead Holter ECG to identify patients with AF-induced cardiomyopathy. In clinical routine this could be a time and cost-efficient discriminator for general practitioners performing Holter ECG to identify patients requiring referral to a cardiologist.

## Clinical Implications

Still, these new technologies require validation in clinical settings and a substantiated choice of the appropriate method using the appropriate device for the patient or user (3, 4). The DoubleCheck-AF validation study from Bacevicius et al. prospectively evaluated a wrist-worn device providing both continuous PPG-based rhythm monitoring and simultaneous 6-lead ECG. The study confirms a high specificity of the underlying algorithm to detect atrial fibrillation and to differentiate atrial fibrillation from other differential diagnoses, like frequent premature contractions.

The optimal screening strategy remains to be found (5). Following important screening trials like the STROKESTOP study (6), current consensus documents extended their recommendations on target populations and settings for screening for atrial fibrillation (3). Furthermore, the implementation of systematic screening for AF to achieve long-term reduction in a combined outcome of mortality, stroke, and severe bleeding is supported by current evidence (7), but will require establishment of clear diagnostic patient pathways.

The DoubleCheck-AF study opens the door for new screening strategies using PPG-based technology as the initial screening device and extending with ECG-based devices in case of irregular pulse notifications Bacevicius et al.. Fabritz et al. present the study design of the investigator-initiated multicenter Smart in OAC – AFNET 9 study which will include 1,000 unselected individuals of 65 years or older on wearable-based screening for PPG-detected atrial arrhythmias.

In post stroke patients, searching for AF is of utmost importance and strongly recommended (3, 8). Wouters, Gruwez, Vranken, Ernon, et al. present a nice case report of a patient simultaneously monitored by an implantable loop recorder and a PPG device.

In preliminary results from the REMOTE trial, Wouters, Gruwez, Vranken, Vanhaen, et al. present their initial results from 39 patients monitored with an implantable loop recorder and a PPG-based device. Interestingly, using the implantable loop recorder as the gold standard compared to a PPG-based monitoring, they identified limitations of the mHealth technology, but also registered false-positive recordings by the implantable loop record requiring revision by a physician.

For patients after cryptogenic stroke, the CANDLE-AF study will clarify the role of a single-lead patch ECG for the early detection of AF (Jung et al.).

In a novel outlook on use of wearables, patients after coronary bypass surgery used a digital device to study the relationship between heart rate variability and pulse rate variability (Chen et al.).

Digital devices not only measure the cardiac rhythm, but can also be used for further risk stratification and clinical decision-making. In a sub-study from the TeleCheck-AF project (9), Hermans et al. analyzed the patient responses to an app-based 10-item questionnaire on risk factors. They found that self-reported mHealth-based assessment of AF risk factors is feasible, but still bears the risk of over- or underreporting. This sets the stage for new approaches to mHealth-based clinical pathways.

## Conclusions and Future Perspectives

Wearable devices for cardiac rhythm monitoring are common. For practical implementation it is key that health care professionals learn about the benefits and pitfalls of new devices, how to interpret the tracings, but also how to integrate this knowledge in practical patient pathways. Further studies are needed to identify the optimal target populations, the best screening settings, establish gold standards, and identify appropriate interventions.

## Author Contributions

DD and ES drafted the work and revised it critically for important intellectual content. Both authors approved publication of the content and agree to be accountable for all aspects of the work in ensuring that questions related to the accuracy or integrity of any part of the work are appropriately investigated and resolved.

## Conflict of Interest

DD has received speaker honoraria and/or travel grants from Abbott, Astra Zeneca, Bayer, Biotronik, Boehringer Ingelheim, Boston Scientific, CVRx, Medtronic, Pfizer, and Zoll. ES has received lecture fees from Bayer, Bristol-Myers Squibb-Pfizer, Boehringer- Ingelheim, Merck Sharp & Dohme, and Sanofi.

## Publisher's Note

All claims expressed in this article are solely those of the authors and do not necessarily represent those of their affiliated organizations, or those of the publisher, the editors and the reviewers. Any product that may be evaluated in this article, or claim that may be made by its manufacturer, is not guaranteed or endorsed by the publisher.
